# The Haitian Health Cluster Experience: A comparative evaluation of the professional communication response to the 2010 earthquake and the subsequent cholera outbreak

**DOI:** 10.1371/5014b1b407653

**Published:** 2012-09-05

**Authors:** Paul Dhillon, Giuseppe Annunziata

**Affiliations:** International Research Lead - Retrieval, Emergency and Disaster Medicine Research and Development Unit (REDSPoT). Saskatchewan, Canada; Chief of Information Knowledge Management, Mediterranean Centre for Health Risk Reduction, World Health Organization

## Abstract

The 2010 Haitian earthquake and consequent Cholera epidemic taxed the already fragile health system. A large number of humanitarian organizations participated in the disaster response and the health communication response was analysed. Health Cluster updates from both periods were analysed for contents with a World Health Organization draft check list for monitoring and evaluating the quality of epidemiological data contained in WHO and Health Cluster emergency reports. The Pan-American Health Organization Emergency Operations Centre reports from the Earthquake had the lowest score with an average score of 2.54/17 and the Health Cluster – Cholera reports had the highest average score of 11/17. There is a wide variety and quality of information published in terms of epidemiological information in emergency reports with a distinct difference found between the earthquake reporting and the cholera event. A comprehensive and modifiable template for emergency reporting could alleviate these differences and allow for improved reporting. Citation: Dhillon P, Annunziata G. The Haitian Health Cluster Experience: A comparative evaluation of the professional communication response to the 2010 earthquake and the subsequent cholera outbreak. PLOS Currents Disasters. 2012 Sep 5. doi: 10.1371/5014b1b407653.

## Introduction

Two disasters in 2010, the Haitian earthquake and the cholera epidemic, caused severe distress on an already fragile health system and impoverished nation to create a unique environment in which comparisons of the public communication response can be analysed.

A complete analysis of the health response and information dissemination through all methods available is beyond the scope of this paper. However a more focused and in depth analysis of specific aspects of the health response can be done. In particular this paper attempts to analyse how the two emergency events were communicated to other professional humanitarian response agents involved in the Health Cluster. In principle, professional communications implies production and dissemination of documents that are usually posted to the internet as the primary means of communication with all stakeholders. The study reviews and compares key papers produced by the Pan-American Health Organization^1^ and the Health Cluster in collaboration with the Ministry of Public Health of Haiti.

This study focuses solely on documents produced by PAHO/WHO and the UN Health Cluster and applies a draft checklist for epidemiological completeness to these documents. This assumes that the writers and organizations that prepared the documents were aware of the importance of epidemiological reporting and were versed in its use and application, it follows that the information was available and could have been present in the reports.

## Background

In 2010, the Repiblik Ayiti (République d’Haïti) was the poorest nation in the Western Hemisphere according to the United Nations Human Development Index.^1^ A population of nine million people, with 67% living on less than $2USD a day were living in cramped conditions.^2^ ^3^ Compounding this fragile baseline was a 7.0 magnitude earthquake, the strongest in recorded history on the fault line, occurring on January 12, 2010. The earthquake shattered infrastructure and estimates of the total loss of life are over 200,000 with more than 300,000 injured.^2^ The lack of enforcement of building codes led to 1.5 million people being made homeless, and one year after this devastation more than 1 million people were still living in camps.^2^

Relief aid flooded into the country from around the world to help rebuild the shattered country. In particular the already sub-standard health care system was greatly affected. Eight hospitals were totally destroyed and 22 were seriously damaged in the three regions most affected.^4^ Prior to the earthquake there were 594 primary health care centres; 30 referral communal hospitals with 30-60 beds each; and 10 department hospitals.^5^

As the country was beginning to rebuild, with the Health Cluster attempting to coordinate the health response to the earthquake, a new threat was emerging which threw the recovery process into further chaos. On October 19, 2010 the first cases of cholera were confirmed on the island nation.^1^

The Health Cluster is a manifestation of a process initiated in 2004 after Jan Egeland began a Humanitarian Response Review to look at ways to better coordinate the humanitarian response at a global level. It is essentially the bringing together of disparate organizations under the umbrella of health in order to better coordinate action in that realm. This strategy has also been employed for other areas as well such as water/sanitation, logistics, and nutrition.

The cholera epidemic added new difficulties just as the health sector was beginning to recover and rebuild. Ten weeks after the start of the epidemic, all departments were affected. This was a surprising event to many as cholera had not been documented in Haiti for decades. On July 25, the Ministry of Health of Haiti (Ministère de la Sante Publique et de la Population, MSPP) reported a total of 388,958 cumulative cholera cases and 5,899 deaths due to cholera. 206,882 patients (53.2%) have been hospitalized out of the total cholera cases.^6^ The two crises in the same nation provide an interesting retrospective comparison between an earthquake and a cholera epidemic, both unexpected and rapidly escalating events that had vastly different levels of staffing on the ground at the onset of each.

**Epidemiological Information Image of Cholera from OCHA:**
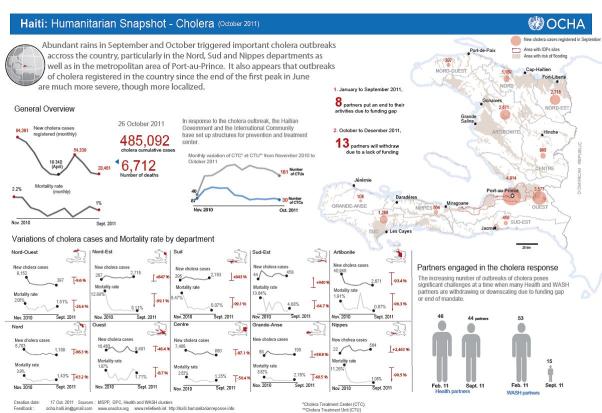
The Health Cluster http://reliefweb.int/sites/reliefweb.int/files/resources/map_1289.pdf

Lessons learned from previous disaster responses have illustrated that humanitarian aid is in some cases wasted or not matched to on the ground needs.^7^ A Humanitarian Response Review (HRR) was commissioned by the Emergency Relief Coordinator (ERC), Jan Egeland, towards the end of 2004. His feelings about the slow and inadequate response in Darfur, Sudan by the humanitarian community signalled weaknesses that needed to be addressed. Four consultants were hired to map the capacity of the humanitarian community at the global level and to suggest changes that would help to ensure a more predictable response to humanitarian crises.

The HRR final report came with a number of recommendations that covered a wide range of issues including human resources, common humanitarian services, coordination, and the idea of creating ‘clusters’ in order to provide greater accountability and predictability in the humanitarian response.

The Health Cluster serves as a mechanism for coordinated assessments, joint analyses, development of agreed overall priorities and objectives, health crises response strategy, and the monitoring, evaluation, implementation, and impact of that strategy. Agencies delivering aid are grouped together in similar areas of expertise and service delivery. The Health Cluster is usually led by the World Health Organization but can be led by multiple organizations. One of the products of the Health Cluster is the Health Cluster Bulletin which is a regular publication drafted in the field and presenting the main health needs and the activities that are ongoing and planned to address those needs.

In the Haitian situation there were 12 clusters in total and the Health Cluster was led by the World Health Organization and the Pan-American Health Organization in close cooperation with the MSPP. Prior to the earthquake the PAHO/WHO staff numbered 52 and more than 60 additional expert staff were added immediately after the earthquake.8, 9 Operational only four days after the earthquake, more than 400 agencies and organizations had registered with the Health Cluster.^10^

The aim of the cluster approach is to improve the coordination and cooperation between the many different response actors while adhering to accepted humanitarian principles.^4^ The health cluster lead was PAHO/WHO, in the initial stages after the earthquake, who worked closely with the Haitian Ministry of Health. They then subsequently became partners in leading the cluster – in some quarters this was deemed one of the largest successes of the coordination aspect of the mission in that the international response organizations goals were appropriately aligned with national structures and long term goals.^4^

An examination of key parts of the Health Cluster Guide provides the theoretical framework for this paper; one function of the Cluster Lead Agency is Monitoring and Reporting and roles are outlined in Table 1 below.

**Table 1. Cluster Function 8:** 

*Cluster Lead Agency*		** *Cluster Partners*
**Cluster Lead Agency Representative**	**Health Cluster Coordinator (HCC)**
Produce and disseminate Cluster sit-reps and regular Health Bulletins using HCC input.	Ensure partners’ active contribution to and involvement in joint monitoring of individual and common plans of action for health interventions; collate and disseminate this and other information related to the health sector in Cluster sit-reps and/or regular Health Bulletins.	Participate in defining and agreeing on any information and reports that Cluster partners should provide to the HCC, and provide such information and reports in a timely manner

*(Modified from Health Cluster Guide, Page 38)

Furthermore the Health Cluster Guide states that the CLA/HCC should bring together all significant health actors and facilitate a process of analyzing available assessment information and agreeing on an initial response strategy for which it is essential to have accurate information disseminated to the different actors involved. This distribution of information is done through the health bulletins and documents posted to the internet.

Drawing from this, the main question this paper addresses is whether or not there were reporting differences in key professional, and publically available, documents distributed during the implementation of the health responses to the Haitian earthquake and Cholera epidemic.

## Methodology

This paper analyses the content of the following document groups which were posted to the internet during the crises;

1. The Pan American Health Organization (PAHO), Health Cluster in Haiti Bulletin, Earthquake;

2. The PAHO/World Health Organization (WHO), Emergency Operations Center Situation Report, Earthquake;

3. PAHO/WHO, Emergency Operations Center Situation Report, Cholera; and

4. PAHO/WHO/Ministere de la Sante Publique et de la Population (MSPP), Health Cluster Bulletin, Cholera and Post-Earthquake Response in Haiti.

Particular attention was be paid to timing of the production of the bulletins and to their content. A set of draft standard references has been generated by the World Health Organization for the function of monitoring and evaluating the quality of epidemiological data published by various WHO and Health Cluster emergency reports and a modified version of these is used to analyse the content of the reports. (Appendix A)

The checklist points were assigned a value of one point for each aspect leading to a maximum value of 17 for any particular bulletin and a minimum value of zero. In addition to scored content the frequency of publication and average page length of each bulletin was ascertained.

This paper is retrospectively examining and comparing the contents and frequency of a segment of the online updates from the Health Cluster during the Haitian cholera and earthquake in 2010. This task included sourcing the documents online and then applying a WHO rubric for evaluating the quality of the epidemiological data that they contained.

All Health Cluster Bulletins and PAHO/WHO Emergency Operations Centre Bulletins were obtained from the Relief Web website and PAHO/WHO web domains.11, 12 One of a total of 91 documents was not available online and was therefore excluded.

**Table 2. Source Documents:** 

Organization/Description	Dates	#
PAHO/WHO Health Cluster Bulletin (Earthquake)	January 20 – March 1, 2010	1-23^*^
PAHO/WHO Emergency Operations Center Situation Report (Earthquake)	January 13 – April 15, 2010	1-26
PAHO/WHO Health Cluster Bulletin (Cholera)	November 11, 2010 – May 27, 2011	1-25
PAHO/WHO Emergency Operations Center Situation Report (Cholera)	October 22 – December 15, 2010	1-17
*Missing #22		

The evaluation framework used was the Health Cluster activities of the WHO. Specifically Function 8, Monitoring and Reporting and Function 9, Advocacy and resource mobilization.^13^

A draft check list for monitoring and evaluating the quality of epidemiological data published by various WHO and Health Cluster emergency reports was provided by Dr. Giuseppe Annunziata, Chief Information and Knowledge Management, WHO Mediterranean Centre for Health Risk Reduction and was used as a comparative tool (Appendix 1) through an Excel comparison template.

**Document Selection and Description**

A total of 90 documents were located and downloaded electronically and subsequently printed. They ranged in size from 2 pages to 16 pages (single sided) and were in Adobe Portable Document Format. A sample cover image of a bulletin is included as Appendix B.

**Data Collection Procedures**

Data was collected by the main investigator and input into Excel. Each document was reviewed twice for accuracy in data collection and a second trained reviewer was used to ensure validity through randomly selected checks of data entry.

**Data Analysis**

The data spread-sheet was then analysed along a number of metrics. The main issue examined was the checklist score of which a maximum value of 17 was possible. Data was plotted on graphs and means were found for the following variables:

a. Average score per bulletin

b. Average days between bulletin production

c. Average length of bulletin

## Results

The average score of the bulletin’s was calculated and is shown below with the PAHO/WHO Emergency Operating Centre receiving an average score of 2.54/17 and the Health Cluster Cholera Bulletin’s receiving the highest value of 11 out of a possible maximum of 17.

**Average Bulletin Score:**
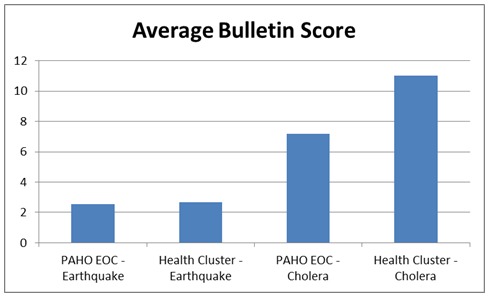

****

In terms of how many days passed between bulletin production the Cholera Health Cluster bulletins were produced every 7.21 days and the Earthquake Health Cluster Bulletins were produced every 2.17 days.

**Bulletin production per day.**
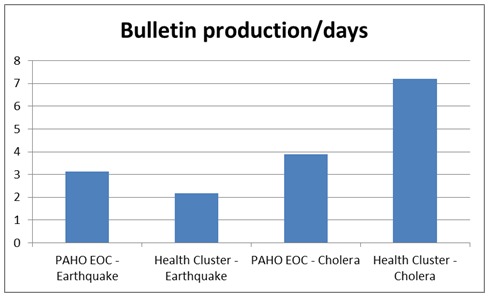

The scores obtained by the Cholera Emergency Operations Centre and the Cholera Health Cluster over time are illustrated below with the Cholera Health Cluster scoring higher than the EOC throughout their production cycle.

**Cholera EOC and Health Cluster Average Score Over Time:**
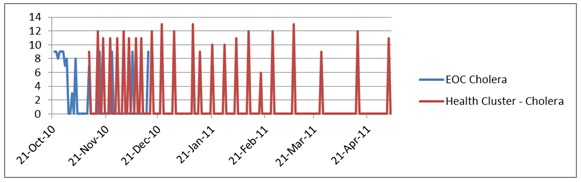

****

The Earthquake Health Cluster and the Earthquake EOC illustrated similar results with the epidemiological score for the Health Cluster higher than the EOC throughout their production.

**Earthquake EOC and Health Cluster Average Score Over Time:**
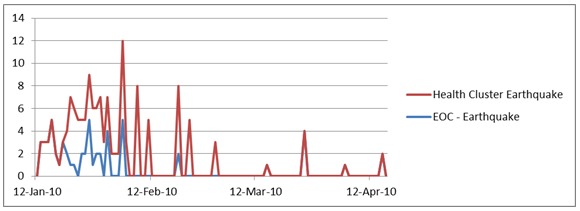

## Limitations

As this is the first time the draft checklist for monitoring and evaluating the quality of epidemiological data in publications by various WHO and Health Cluster emergency report’s was completed there is currently no way to validate the results or compare them against previous studies. An online review does not illustrate that there are previous standardized templates for reporting across the different bodies that produced the reports and the aims of the emergency bulletins are not inherently clear. As was noted on the front page of one of the bulletin’s late in its production cycle, there were questions about the contents and relevance of the reports.

The major limit to this data is that for all intents and purposes it is the first to be analysed using the WHO Checklist for quality of epidemiological data in the comparison of two events in the same country. However, future studies that utilize the same checklist can ascertain the validity of this study. Another major limitation is the lack of standardization of the different health bulletins; there are multiple opportunities where interpretation of the presence or absence of quantitative variables could have an impact on the final results.

The study was limited by the number of bulletins that were published online and this was required in order to be able to complete the study in a location remote from Haiti and in a retrospective fashion that is reproducible in future studies.

## Discussion

One of the goals of this paper was to compare the response to two distinct disasters within the same country. In particular, by focusing on the epidemiological data published in key documents for professional communication it became readily apparent that emphasis in the bulletins was placed more on epidemiological information during the cholera outbreak in comparison to the earthquake. This was illustrated in Figure 2 with the starkly higher average scores during the cholera outbreak in comparison to the earthquake. However, this could be heavily influenced by the fact the infrastructure on the ground was severely limited in the aftermath of the earthquake and there was much more plentiful staff on the ground during the cholera epidemic.

Throughout the reporting, when looking longitudinally across the time of the report generation, it was seen that the Health Cluster scores for each bulletin in terms of epidemiological data was higher than in the Emergency Operations Centre bulletins; this could be due to a number of different factors. One plausible factor could be the intended market audience for the reporting; at no stage was there a direct indication of who the reports were addressed to. Assuming they were for the organizations working in each of the disasters as well as for greater public dissemination, including the media, there was perhaps too broad of a market for the information creating a dilutional effect of what was included and what was not. In fact, in one of the later bulletins, it was specifically mentioned that the goals of the bulletin were being re-evaluated in order to more closely address the needs of the readership. There was likely a strong amount of overlap between the EOC reports and the Health Cluster Bulletin’s and this is an area in which duplication of work could be reduced with a closer integration of the two bodies.

## Conclusions and Recommendations

What this review of professional organizations published emergency bulletin’s found was that there is a wide variety and quality of information published in terms of epidemiological data in emergency reports. In the earthquake reporting, a sudden onset ‘non-medical’ emergency, there is an apparent lack of epidemiological data present throughout the reports. In comparison, there is more epidemiological notation, but varied information in the more ‘medical’ cholera epidemic reporting.

Closer collaboration between professional organizations should be strived for within disaster medicine when deciding which metrics are required for complete epidemiological reporting. A comprehensive template, modifiable for different situations with key metrics always including could be used in order to standardize reporting globally. This would allow for more research into professional communication outcomes and comparison across nations and disasters in addition to allowing for more accurate communication to donors and the greater public through the media.

## Competing Interests

Dr. Paul Dhillon has no competing interests in the production of this paper. Dr Giuseppe Annunziata was employed at the Chief Information and Knowledge Management Office, WHO Mediterranean Centre for Health Risk Reduction during the production of this paper.

## Appendix A

### Check list for monitoring and evaluating the quality of epidemiological data published by various WHO and Health Cluster emergency reports – DRAFT

Epidemiological data published on Health Cluster Bulletins and/or WHO Emergency Situation Reports should include:

1. Location of collected data: country, state, province, district, city or village

2. Timing: date of reporting ; period of reporting (from/to)

3. Organization reporting: WHO, Health Cluster, etc.

4. Source of information: MoH, WHO, EWAR, NGOs, rumours, media.

5. Identification of the event reported: name according to ICD-10; case definition adopted in the context (annex)

6. Number of new cases: suspected vs confirmed with lab test; age class/gender; by time period (by day, week, month according to the seriousness of the event)

7. Incidence or proportional morbidity by time period

8. Attack rate (cumulative incidence)

9. Number of deaths: by age class/gender; by time period; case fatality ratio by time period

10. Graphs: incidence or proportional morbidity by line graphs, absolute number of cases by histograms

In addition to the below points, epidemiological data published on Emergency Weekly Epidemiological Bulletins produced by MoH, WHO and/or Health Cluster partners should include:

11. Surveillance system: sentinel/exhaustive; type of facilities

12. Completeness: % of facilities reporting by time period and location

13. Timeliness: % of facilities reporting on time by time frame and location

14. Events: list of the events under surveillance; priority to epidemic prone diseases with potential high health impact and whose prevention and control is possible; include “other” category to calculate proportional morbidity.

15. Alerts: number per week by location; number investigated by whom

16. Laboratory: number of samples taken and location; number of positive samples

17. Graphs: completeness of reporting by line graphs

[1] The Pan American Health Organization (PAHO) is an international public health agency with over 100 years of experience working to improve health and living standards of the people of the Americas. It enjoys international recognition as part of the United Nations system, serving as the Regional Office for the Americas of the World Health Organization, and as the health organization of the Inter-American System.
